# Responses of *Aspergillus flavus* to Oxidative Stress Are Related to Fungal Development Regulator, Antioxidant Enzyme, and Secondary Metabolite Biosynthetic Gene Expression

**DOI:** 10.3389/fmicb.2016.02048

**Published:** 2016-12-21

**Authors:** Jake C. Fountain, Prasad Bajaj, Spurthi N. Nayak, Liming Yang, Manish K. Pandey, Vinay Kumar, Ashwin S. Jayale, Anu Chitikineni, Robert D. Lee, Robert C. Kemerait, Rajeev K. Varshney, Baozhu Guo

**Affiliations:** ^1^Department of Plant Pathology, University of GeorgiaTifton, GA, USA; ^2^United States Department of Agriculture, Agricultural Research Service Crop Protection and Management Research UnitTifton, GA, USA; ^3^International Crop Research Institute for the Semi-Arid TropicsHyderabad, India; ^4^Department of Crop and Soil Sciences, University of GeorgiaTifton, GA, USA

**Keywords:** *Aspergillus flavus*, aflatoxin, aflatrem, kojic acid, oxidative stress

## Abstract

The infection of maize and peanut with *Aspergillus flavus* and subsequent contamination with aflatoxin pose a threat to global food safety and human health, and is exacerbated by drought stress. Drought stress-responding compounds such as reactive oxygen species (ROS) are associated with fungal stress responsive signaling and secondary metabolite production, and can stimulate the production of aflatoxin by *A. flavus in vitro*. These secondary metabolites have been shown to possess diverse functions in soil-borne fungi including antibiosis, competitive inhibition of other microbes, and abiotic stress alleviation. Previously, we observed that isolates of *A. flavus* showed differences in oxidative stress tolerance which correlated with their aflatoxin production capabilities. In order to better understand these isolate-specific oxidative stress responses, we examined the transcriptional responses of field isolates of *A. flavus* with varying levels of aflatoxin production (NRRL3357, AF13, and Tox4) to H_2_O_2_-induced oxidative stress using an RNA sequencing approach. These isolates were cultured in an aflatoxin-production conducive medium amended with various levels of H_2_O_2_. Whole transcriptomes were sequenced using an Illumina HiSeq platform with an average of 40.43 million filtered paired-end reads generated for each sample. The obtained transcriptomes were then used for differential expression, gene ontology, pathway, and co-expression analyses. Isolates which produced higher levels of aflatoxin tended to exhibit fewer differentially expressed genes than isolates with lower levels of production. Genes found to be differentially expressed in response to increasing oxidative stress included antioxidant enzymes, primary metabolism components, antibiosis-related genes, and secondary metabolite biosynthetic components specifically for aflatoxin, aflatrem, and kojic acid. The expression of fungal development-related genes including aminobenzoate degradation genes and conidiation regulators were found to be regulated in response to increasing stress. Aflatoxin biosynthetic genes and antioxidant enzyme genes were also found to be co-expressed and highly correlated with fungal biomass under stress. This suggests that these secondary metabolites may be produced as part of coordinated oxidative stress responses in *A. flavus* along with antioxidant enzyme gene expression and developmental regulation.

## Introduction

The contamination of crops with aflatoxin, a carcinogenic secondary metabolite of the facultative plant parasite *Aspergillus flavus* (Guo et al., 1996), is a threat to human health, global food safety and security (Williams et al., 2010; Guo et al., 2012; Torres et al., 2014; Andrade and Caldas, 2015). Aflatoxin contamination of staple and dietary supplemental crops such as maize and peanut result in both losses in crop value in international trade due to restrictions on aflatoxin content (Matumba et al., 2015; Wu, 2015), and negative impacts in human and animal health (Williams et al., 2004, 2010; Kew, 2013). These concerns are the impetus for investigations into the biology of this organism and its interactions with host plants related to aflatoxin contamination (Diener et al., 1983, 1987; Amaike and Keller, 2011; Guo et al., 2012; Fountain et al., 2014).

The aflatoxin biosynthetic pathway has been well characterized in *A. flavus* and in other aflatoxigenic species of *Aspergillus* such as *A. parasiticus*, and sterigmatocystin producing species such as *A. nidulans* (Amaike and Keller, 2011). Aflatoxin biosynthesis is encoded by a cluster of 25 genes which has been highly conserved among *Aspergillus* spp. and has been well characterized (Yu et al., 2004). While the biosynthetic mechanisms involved in aflatoxin production have been well characterized, little is known regarding the biological role of aflatoxin in *A. flavus* or other *Aspergillus* spp. Secondary metabolites produced by soil-dwelling fungi exhibit various biological activities including fungivory resistance, stress tolerance, and quorum sensing (Reverberi et al., 2008, 2010; Roze et al., 2013).

Recent studies have shown that reactive oxygen species (ROS) and their reactive products such as peroxidized lipids (oxylipins) are required for the production of aflatoxin and can stimulate aflatoxin production if applied *in vitro* (Jayashree and Subramanyam, 2000). Induction of oxylipin and ROS accumulation in *A. flavus* mycelia through peroxisome proliferation has also been linked with increased aflatoxin production and antioxidant enzyme activity (Reverberi et al., 2012). Similarly, several studies have also been performed examining the effects of antioxidants on the growth and aflatoxin production of Aspergilli. For example, phenolic compounds such as caffeic acid tannic acid derived from tree nuts have been shown to inhibit aflatoxin production in *A. flavus* (Mahoney et al., 2010). Other synthetic phenolic compounds such as butylated hydroxyanisole (BHA) and propyl paraben (PP) have also been found to have a similar effect as a function of medium pH and water activity (Nesci et al., 2003; Passone et al., 2005). Treatment with BHA as also been shown to inhibit sclerotial differentiation in *A. flavus*. Oxidative stress responsive signaling mechanisms have been found to be involved in the regulation of aflatoxin production such as the stress responsive transcription factors AtfB, AP-1, and VeA (Reverberi et al., 2008; Sakamoto et al., 2008; Roze et al., 2011, 2013; Hong et al., 2013a,b; Baidya et al., 2014). Also, VeA along with VelB and LaeA form the Velvet protein complex to regulate both secondary metabolite production and reproductive development in *A. flavus* and other *Aspergillus* spp. (Park et al., 2012). The consumption and/or generation of ROS have also been found to co-localize to vesicles known as aflatoxisomes where the final phases of aflatoxin production are carried out (Chanda et al., 2009, 2010; Roze et al., 2015).

Because of the close association of ROS and aflatoxin production, it has been hypothesized that aflatoxin production may serve as a component of oxidative stress alleviation mechanisms employed by *Aspergillus* spp. in addition to antioxidant enzymes, altered carbon metabolism, and the production of other secondary metabolites (Narasaiah et al., 2006; Roze et al., 2013; Fountain et al., 2014, 2016). The tolerance of *A. flavus* and *A. parasiticus* isolates to oxidative stress has been shown to be correlated with their levels of aflatoxin production. For example, Roze et al. (2015) showed that conidia of isolates with higher levels of aflatoxin production also exhibited greater viability when cultured in ROS-amended medium.

This correlation between ROS and aflatoxin production has also lead to the hypothesis that host plant-derived ROS and oxylipins may function in the host-parasite interaction between *A. flavus* or other Aspergilli and their host plants. Host plant resistance to aflatoxin contamination have been identified, and this resistance has been found to be heavily influenced by environmental stresses, particularly drought stress (Diener et al., 1983, 1987; Williams, 2006; Guo et al., 2008; Holbrook et al., 2009; Jiang et al., 2012; Kebede et al., 2012; Pandey et al., 2012; Fountain et al., 2014). Interestingly, these aforementioned ROS and oxylipins have also been shown to accumulate in the tissues of host plants during drought stress, and their levels have been correlated with aflatoxin contamination resistance (Gao et al., 2009; Yang et al., 2015, 2016). Host plant tissue antioxidant enzyme activity and capacity has also been found to be correlated with reduced *A. flavus* growth and aflatoxin production in model species such as buckwheat (Chitarrini et al., 2014).

In our previous studies, isolates of *A. flavus* were found to exhibit different degrees of oxidative stress tolerance which appeared to correlate with their aflatoxin production capability suggesting that aflatoxin production may contribute to stress tolerance (Fountain et al., 2015). However, the observed stress tolerance of the toxigenic isolates cultured in aflatoxin non-conducive medium was reduced yet comparable indicating that factors in addition to aflatoxin production also contributed to the observed differences. In order to better understand the differences in isolate-specific responses to oxidative stress, and to further explore the potential role of aflatoxin production in stress alleviation in *A. flavus*, we examined the global transcriptional responses of several isolates of *A. flavus* to increasing oxidative stress, which has been summarized in an overview-type publication (Fountain et al., 2016). Here, we present a detailed analysis of changes in the transcriptomes of different toxigenic *A. flavus* isolates with distinguished aflatoxin production capabilities to increasing oxidative stress in an aflatoxin conducive culture medium. By examining the oxidative stress responses of *A. flavus*, the biological role of aflatoxin production in stress responses and in competition with other soil-dwelling organisms may be better understood.

## Materials and methods

The methodologies presented here are adapted from Fountain et al. (2016) and describe the data generation and analysis procedures for the toxigenic isolates examined in this study.

### Isolate culture conditions

The *A. flavus* toxigenic isolates utilized in this study were obtained as previously described (Fountain et al., 2015). The isolates AF13, NRRL3357, and Tox4 were initially cultured on V8 agar (20% V8, 1% CaCO_3_, 3% agar) at 32°C for 5 days. Conidia were collected from the cultures by washing the plates with sterile 0.1% (v/v) Tween 20. This conidial suspension (~4.0 × 10^6^ conidia/mL) was used to inoculate liquid cultures of yeast extract sucrose (YES; 2% yeast extract, 1% sucrose) medium containing various concentrations of hydrogen peroxide (H_2_O_2_, 3% stabilized solution). For AF13 and Tox4, the YES medium was supplemented with 0, 10, and 25 mM H_2_O_2_ representing a control, moderately high, and high levels of stress, respectively. For NRRL3357, the YES medium was supplemented with 0, 10, and 20 mM H_2_O_2_ due to the lower concentration of H_2_O_2_ the isolate could tolerate (Fountain et al., 2015). The experiment was performed in 125 mL Erlenmeyer flasks containing 50 mL H_2_O_2_ amended YES medium and 100 μL conidial suspension plugged with a sterile cotton ball. The cultures were incubated at 32°C for 7 days in the dark with two biological replicates for each isolate/treatment combination. Mycelia were then harvested from each culture, immediately frozen in liquid nitrogen, and stored at −80°C.

### RNA isolation

The harvested mycelia were homogenized in a chilled mortar and pestle to a fine powder, and then used for total RNA isolation. Total RNA was isolates using an RNeasy Plant Mini Kit with DNase digestion according to the manufacturer's instructions (Qiagen, Hilden, Germany). The isolated RNA was then quantified using a Nano-Drop ND1000 spectrophotometer (Thermo Scientific, Wilmington, DE, USA) and the RNA integrity numbers (RINs) for each sample were validated using an Agilent 2100 Bioanalyzer (Agilent, Santa Clara, CA, USA). Samples with an RIN ≥ 5 were used for RNA sequencing.

### Library construction and illumina sequencing

The cDNA libraries for each sample and biological replicate were generated from 1 μg of total RNA. A total of 18 libraries were generated for the toxigenic *A. flavus* isolate samples and used for transcriptome sequencing using an Illumina TruSeq RNA Sequencing Kit according to the manufacturer's instructions (Illumina, San Diego, CA, USA). Following quantitation using a Qubit 2.0 fluorometer (Thermo Scientific), and validation using an Agilent 2100 Bioanalyzer (Agilent), the libraries were used for cluster generation using a cBot (Illumina), and paired-end sequencing using a HiSeq 2500 platform according to the manufacturer's instructions (Illumina).

### Bioinformatics analysis

Initial quality checks on raw sequencing reads were performed using FastQC v0.11.2 with low quality reads being removed using Trimmomatic v0.32, along with rRNA contamination following alignment with the SILVA database. The remaining, high quality reads were used for differential expression analysis using the tuxedo protocol. Briefly, alignment of filtered reads to the *A. flavus* NRRL3357 reference genome (GCF_000006275.2) was done using tophat2 v2.0.13 and bowtie2 v2.2.4. Cufflinks v2.2.1 and cuffdiff were used to assemble the transcripts and determine transcript abundance in terms of Fragments Per Kilobase of exon per Million fragments mapped (FPKM). A gene was considered significantly differentially expressed when |log_2_(fold change)| ≥ 2 with an adjusted *p* ≤ 0.05. Tophat2 alignments were used to perform RABT (reference annotation based transcript) assembly for both genes and isoforms. The assemblies were then compared and merged using cuffmerge and used for analysis.

The assembled transcripts were annotated using a standalone blast 2.2.30+ and analyzed through Blast2GO and KEGG for gene ontology (GO) and pathway analysis, respectively. Heatmaps of select secondary metabolites biosynthesis genes and principal components analysis (PCA) of the gene expression profiles of the isolates was performed with multiple experiment viewer (MeV) v4.9.0. For promoter analysis of select genes, upstream gene sequences containing conserved transcription factor binding domains were obtained using FungiDB (http://fungidb.org/) followed by sequence analysis using MEME 4.11.2 (Bailey et al., 2009; Stajich et al., 2012). Co-expression analysis was performed using the weighted correlation network analysis (WGCNA) package in R v3.3.0 (Langfelder and Horvath, 2008).

### Quantitative RT-PCR validation

In order to validate the RNA sequencing results, we selected several genes for expression analysis on a subset of samples using quantitative RT-PCR (qPCR). Using total RNA remaining following library construction, we synthesized cDNA using a Taqman reverse transcriptase kit (Thermo-Fisher) according to the manufacturer's instructions. Reverse transcription was carried out using a PTC-200 thermal cycler (Bio-Rad, Hercules, CA, USA) with the following cycling parameters: 25°C for 10 min, 48°C for 30 min, and 95°C for 5 min. The qPCR was then performed using a 20 μl reaction volume containing: 1X SYBR Green PCR Master Mix (Thermo-Fisher), 0.4 μM forward primer, 0.4 μM reverse primer, and 25 ng cDNA template. In this study, β-tubulin was used as a housekeeping control, and primer sequences for this and the other genes examined can be found in Table S1. The reactions were carried out using an ABI 7500 platform (Thermo-Fisher) using the following cycles: 50°C for 2 min, 95°C for 10 min, and 40 amplification cycles of 95°C for 15 s, and 60°C for 1 min. Dissociation curves were performed at the end of each cycle to identify possible primer dimerization or off-target amplification. With the obtained threshold cycle (Ct) values, relative gene expression was calculated using a modified Livak method where Relative Expression = 2^ΔCt^ and ΔC_t_ = C_t_ Housekeeping Gene (β-tubulin)—C_t_Target (Livak and Schmittgen, 2001).

## Results

### Transcriptome sequencing

In order to examine the transcriptional responses of aflatoxigenic isolates of *A. flavus* to oxidative stress, we cultured three isolates (AF13, NRRL3357, and Tox4) in aflatoxin production-conducive medium amended with H_2_O_2_ at different concentrations. Whole transcriptome sequencing of these tissues yielded a total of 1.32 billion raw sequencing reads from 18 cDNA libraries with an average of 73.17 million reads per library. Of the 18 libraries sequenced, one contained a relatively low number reads and was excluded from the analysis. Initial quality filtration resulted in the removal of 9–10% of reads followed by further removal of rRNA contamination. Following filtration, a total of 687.33 million filtered reads were obtained with an average of 40.43 million reads per library. For all the libraries, an average of 92.64% of filtered reads mapped to the *A. flavus* NRRL3357 reference genome. For the individual isolates, an average of 91.66, 94.52, and 91.58% of filtered reads from AF13, NRRL3357, and Tox4, respectively, mapped to the reference genome.

**Figure 1:**
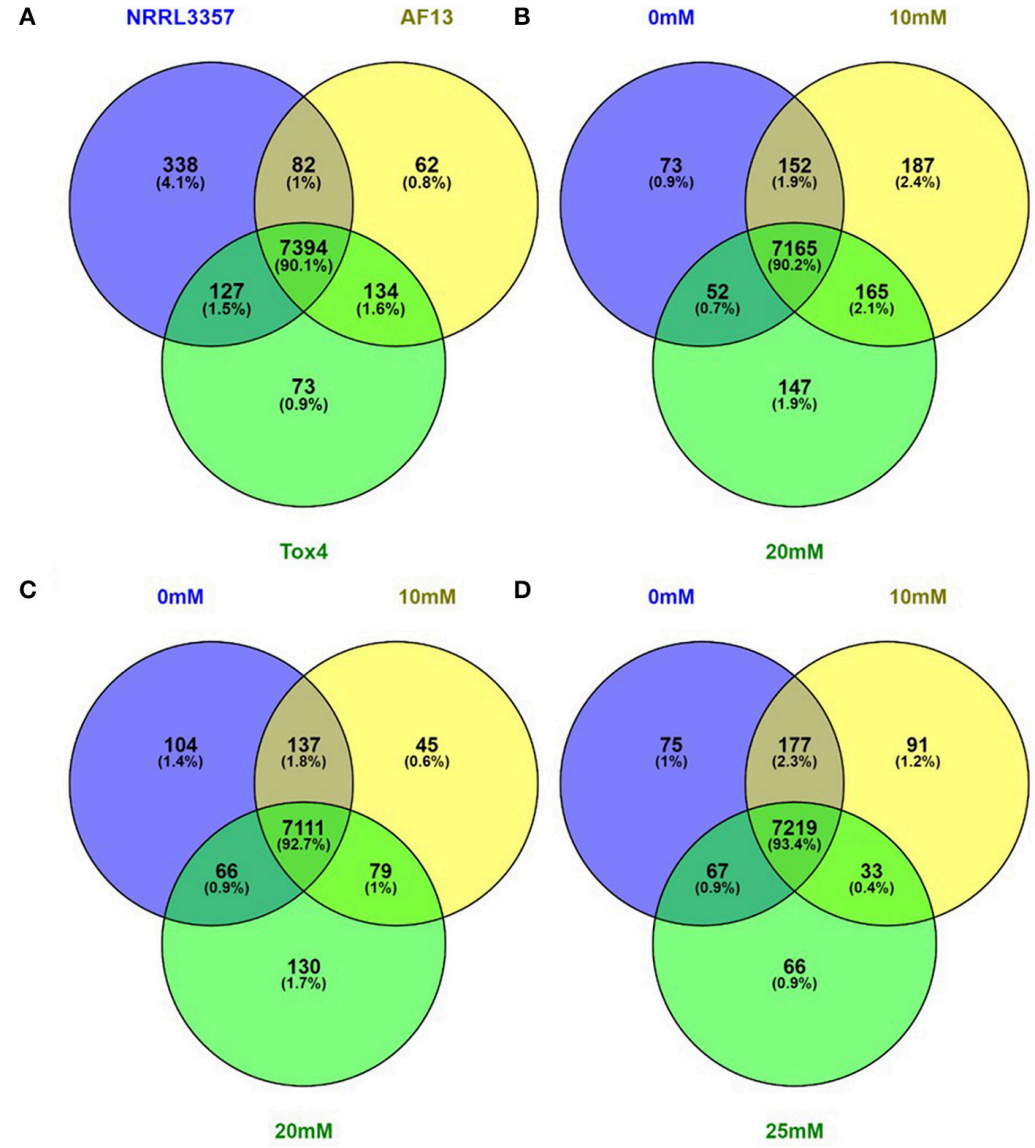
**Venn diagrams of genes expressed with an FPKM ≥ 2**. Genes exhibiting an FPKM ≥ 2 in at least one isolates and treatment were considered to be expressed. Genes expressed within all isolates and treatments **(A)**, and between treatments within the NRRL3357 **(B)**, AF13 **(C)**, and Tox4 **(D)** isolates were compared using Venn diagrams generated with Venny 2.1.0.

Of the 13,487 genes annotated in the NRRL3357 genome, 11,144 genes (82.63%) were described in the dataset. Among those described genes, 8210 genes (73.67%) of the genes had an FPKM ≥ 2 in at least one isolate and treatment (Dataset S1). Of these genes with an FPKM ≥ 2, 338 (4.12%), 62 (0.76%), and 73 (0.89%) genes were exclusively expressed in NRRL3357, AF13, and Tox4, respectively, across all treatments examined in the study (Figure 1A). For each individual isolate, a relatively low number of genes were exclusively expressed with an FPKM ≥ 2 within a specified treatment. For NRRL3357, 73 (0.92%), 187 (2.35%), and 147 (1.85%) of the 7941 genes expressed were exclusive to the 0 mM, 10 mM, and 20 mM H_2_O_2_ treatments, respectively (Figure 1B). For AF13, 104 (1.36%), 45 (0.59%), and 130 (1.69%) of the 7672 genes expressed were exclusive to the 0 mM, 10 mM, and 20 mM H_2_O_2_ treatments, respectively (Figure 1C). For Tox4, 75 (0.97%), 91 (1.18%), and 66 (0.85%) of the 7728 genes expressed were exclusive to the 0 mM, 10 mM, and 20 mM H_2_O_2_ treatments, respectively (Figure 1D). A full list of the uniquely expressed genes in each isolate or treatment can be found in Dataset S2.

### Differential expression is correlated with oxidative stress tolerance

Following transcriptome assembly and alignment, differential expression analyses were performed to examine the oxidative stress responses of the isolates. Genes were considered significantly differentially expressed if they exhibited a |log_2_ (fold change)| ≥ 2 and adjusted *p* ≤ 0.05. The Tox4 isolate exhibited 4 and 29 DEGs when comparing the control with 10 mM H_2_O_2_ and 25 mM H_2_O_2_ treatments, respectively, and 57 when comparing the two treatments (Table 1). The AF13 isolate exhibited 6 and 122 DEGs when comparing the control with 10 mM H_2_O_2_ and 25 mM H_2_O_2_ treatments, respectively, and 85 when comparing the two treatments (Table 1). The NRRL3357 isolate exhibited 53 and 117 DEGs when comparing the control with 10 mM H_2_O_2_ and 20 mM H_2_O_2_ treatments, respectively, and 112 when comparing the two treatments (Table 1).

**Table 1:** **Numbers of significantly, differentially expressed genes**.

**Isolat**	**Toxin^a^**	**H_2_${\text{O}}_{2}^{\text{a}}$**	**0 v 10 mM^b^**	**0 v 20/25 mM^b^**	**10 v 20/25 mM^b^**
Tox4	+++	40	4	29	57
AF13	+++	35	6	122	85
NRRL3357	+	20	53	177	122

a *Aflatoxin production capability (+++, high; +, moderately high) and maximum H_2_O_2_ tolerance observed in Fountain et al. (2015)*.

b *Numbers of DEGs described in Fountain et al. (2016)*.

**Figure 2:**
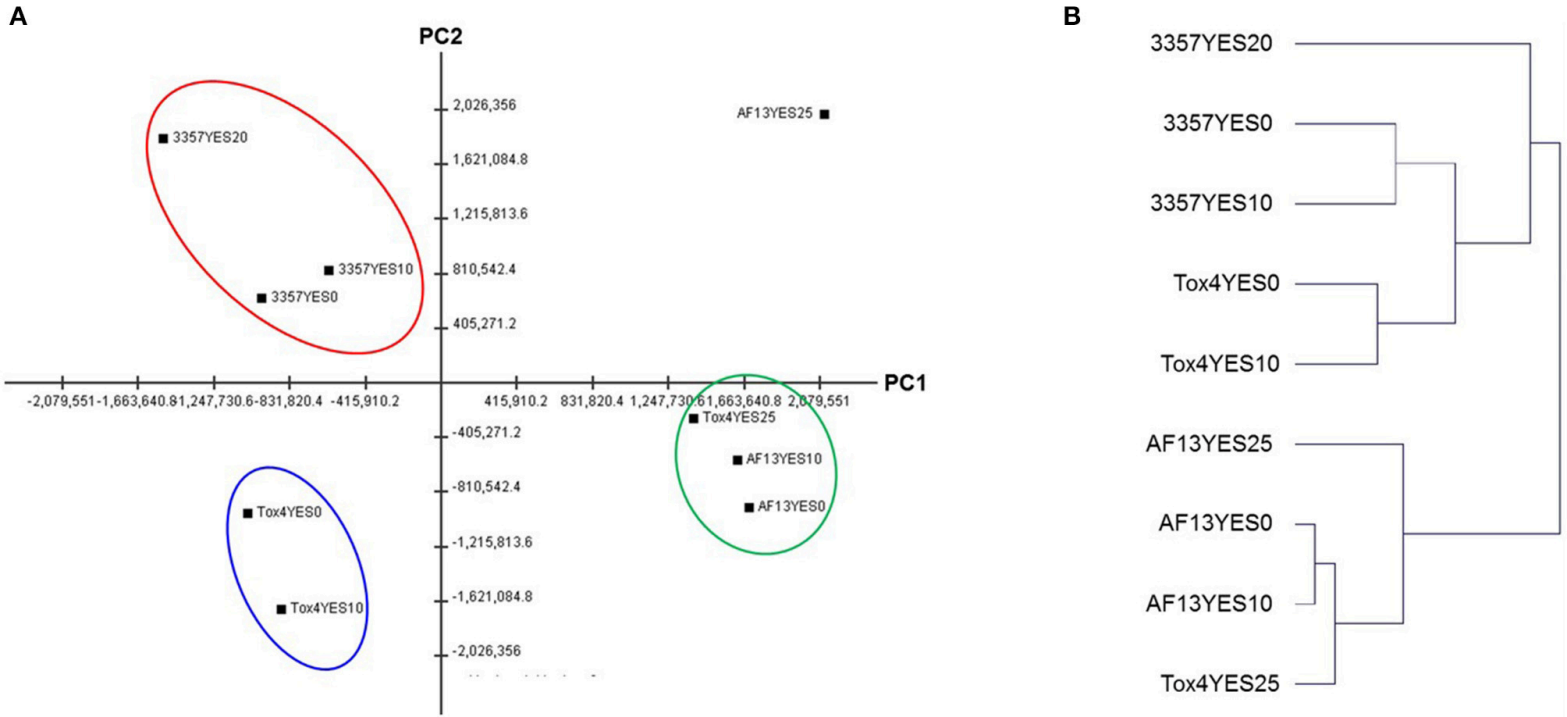
**Principal component analysis (PCA) and hierarchical clustering analysis (HCA) of the isolate gene expression profiles. (A)** Principal component analysis (PCA) of the isolate expression patterns with PC1 and PC2 contributing 33.57 and 25.98% of overall variance, respectively. Similar clusters are indicated by the colored circles. **(B)** Hierarchical clustering analysis (HCA) of the isolate expression patterns. Both analyses indicate a similarity in the expression profiles of AF13 and highly stressed Tox4 with NRRL3357 exhibiting a more distinct profile.

The numbers of significant DEGs for the isolates were then correlated with the previously observed maximum H_2_O_2_-induced oxidative stress tolerance levels observed for each isolate in our previous study (Fountain et al., 2015). The number of DEGs exhibited a strong, negative correlation with the previously observed tolerance levels (Figure S1) when comparing the control and the 10 mM H_2_O_2_ treatment (*r* = −0.979), and when comparing the two treatment conditions (*r* = −0.958). The correlation was not as strong, however, when comparing the control and the 20 or 25 mM H_2_O_2_ treatment (*r* = −0.658). Interestingly, the isolates which exhibited the lower numbers of differentially expressed genes, especially under 10 mM H_2_O_2_, also tended to produce higher levels of aflatoxin (Table 1; Fountain et al., 2015). However, while the isolate with the highest level of H_2_O_2_tolerance, Tox4, did exhibit the fewest number of DEGs, it produces similar levels of aflatoxin to AF13 which exhibited slightly lower H_2_O_2_ tolerance and almost four times the number of DEGs at 25 mM H_2_O_2_ (Table 1). In addition, comparison of the overall expression profiles shows a clear segregation by isolate indicative of the differences in isolate-specific gene expression patterns due to background expression differences between isolates and responses to increasing stress (Figure 2, Table S2).

### Secondary metabolite gene expression is regulated in response to oxidative stress

**Figure 3:**
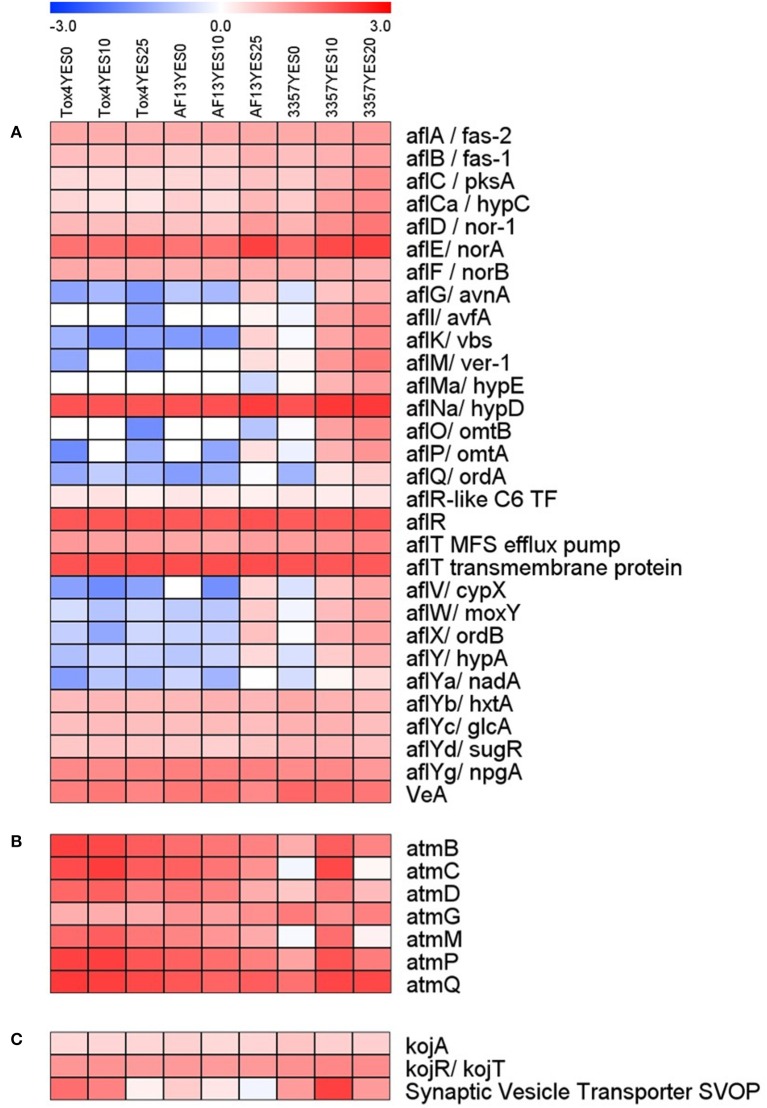
**Heatmap of aflatoxin, aflatrem, and kojic acid biosynthetic gene expression with increasing oxidative stress**. The expression of aflatoxin **(A)**, aflatrem **(B)**, and kojic acid **(C)** biosynthetic genes are plotted with colors corresponding to the level of expression of the genes in each condition. Here, FPKM expression values were log_10_ transformed and plotted based on the color key increasing from blue to red using MeV 4.9. Isolate and treatment combinations for each column are consistent for each heatmap.

Genes involved in the biosynthesis of aflatoxin were differentially expressed in the toxigenic isolates with distinguished aflatoxin production capabilities in response to increasing levels of oxidative stress (Figure 3A). The most significant changes in expression mainly came in the isolate with the least tolerance to high levels of H_2_O_2_ than the other isolates included in this study, NRRL3357, which also produces only a moderate level of aflatoxin under elevated oxidative stress (Fountain et al., 2015). In NRRL3357, the genes encoding O-methyltransferase A (omtA), versicolorin B synthase (vbs), and versicolorin dehydrogenase/ketoreductase (ver-1) were exhibited log_2_(fold change) > 3.61 when comparing 0 and 10 mM H_2_O_2_ treatments, and > 4.85 when comparing 0 and 20 mM H_2_O_2_ (Dataset S3). Additional genes encoding polyketide synthase (pksA), norsolorinic acid ketoreductase (nor-1), and a cytochrome p450 monooxygenase (aflV/cypX) also exhibited log_2_(fold change) > 2.32 when comparing the 0 and 20 mM H_2_O_2_ treatments (Dataset S3). In AF13, the *cypX, omtA*, and *ver-1* genes along with the genes encoding the versicolorin B desaturase p450 monooxygenase (verB), and the regulatory transcription factor aflS/aflJ were upregulated when comparing the 0 and 20 mM H_2_O_2_ treatments (Dataset S4). None of these genes were found to be differentially expressed when comparing 0 and 10 mM H_2_O_2_ (Dataset S4).

Surprisingly, in Tox4, no aflatoxin biosynthesis transcripts were found to be differentially expressed in either treatment (Dataset S5). Among the aflatoxin biosynthetic genes, the averufin p450 monooxygenase (*avfA*), *hypE* hypothetical protein, O-methyltransferase B (*omtB*), oxidoreductase A (*ordA*), and NADH oxidase (*nadA*) genes were expressed with an FPKM ≥ 2 only in NRRL3357 (Dataset S2). The aflatoxin genes encoding averantin p450 monooxygenase (avnA), vbs, ver-1, omtA, cypX, the p450 monooxygenase moxY, oxidoreductase B (ordB), and the hypothetical protein hypA were expressed with an FPKM ≥ 2 only in AF13 and NRRL3357 (Dataset S2). The remaining aflatoxin pathway genes were found to be expressed in AF13, NRRL3357, and Tox4 (Dataset S2).

In addition to aflatoxin biosynthesis genes, genes involved in the biosynthesis of two additional secondary metabolites, aflatrem and kojic acid, were found to be differentially expressed in response to increasing oxidative stress (Figure 3B). In NRRL3357, the aflatrem biosynthesis genes encoding prenyl transferase (atmC) and geranylgeranyl diphosphate synthase (atmG) (Nicholson et al., 2009) were significantly upregulated in when comparing 0 and 10 mM H_2_O_2_ (Dataset S3). The *atmG* gene was however, not upregulated when comparing 0 and 20 mM H_2_O_2_, though it is significantly reduced when comparing 10 and 20 mM H_2_O_2_ indicating a dose-dependent response (Dataset S3). In contrast, in AF13 the dimethylallyl tryptophan synthase gene (*atmD*), *atmC*, and *atmG* were downregulated when comparing 0 and 25 mM H_2_O_2_ while no aflatrem biosynthesis genes were significantly differentially expressed when comparing 0 and 10 mM H_2_O_2_ (Dataset S4). Tox4, however, showed no significant differences in aflatrem biosynthesis gene expression when comparing the control with either treatment, though comparing 10 and 25 mM H_2_O_2_ did show a significant reduction in atmG expression (Dataset S5).

**Figure 4:**
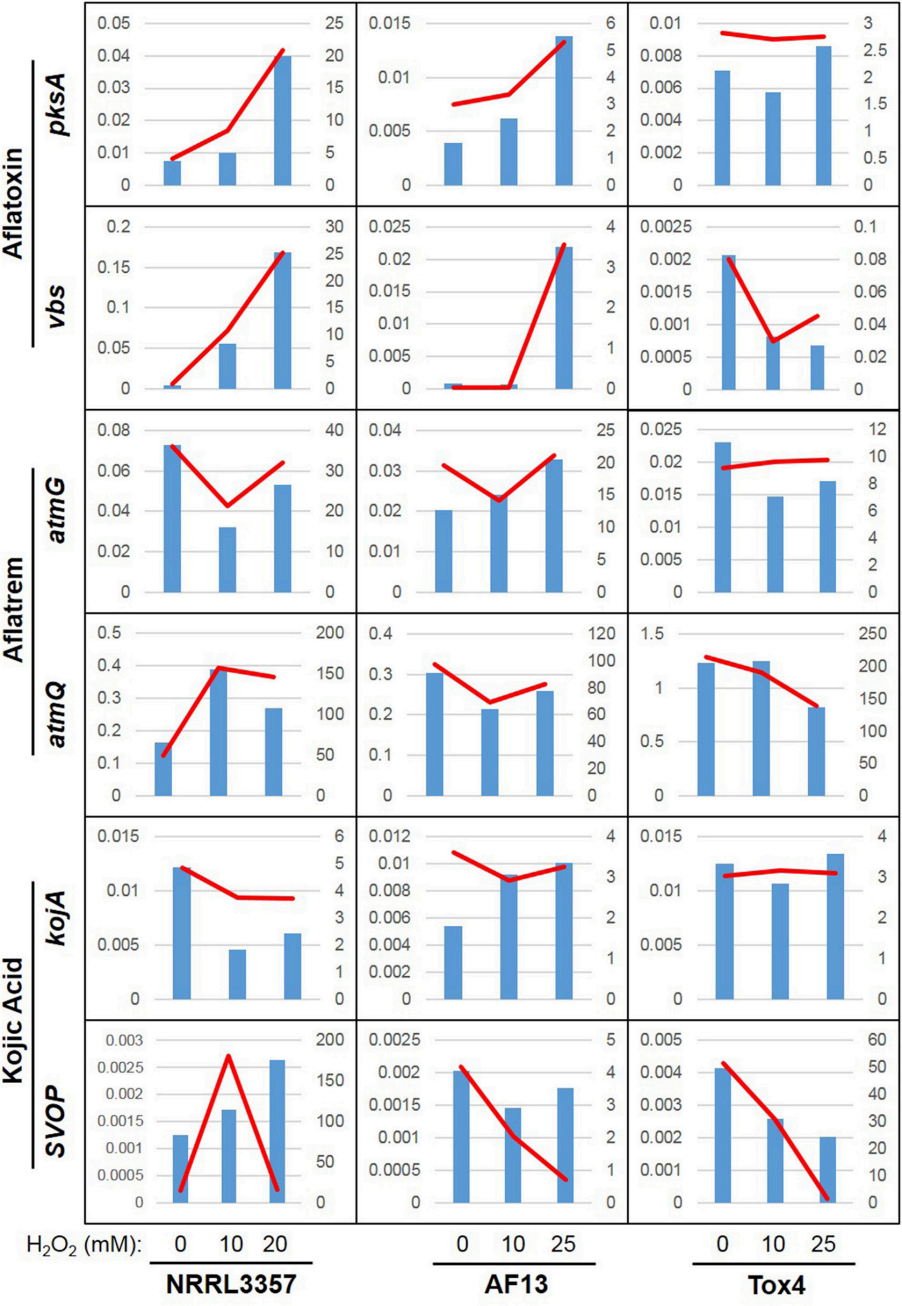
**Relative expression and FPKM associations for select secondary metabolite genes**. Real-time PCR was used to determine the relative expression of six select secondary metabolite genes: *pksA* and *vbs* (aflatoxin), *atmG* and *atmQ* (aflatrem), and *kojA* and *SVOP* (kojic acid). Relative expression is indicated by the blue bars scaled to the left y-axis. The RNA sequencing-derived FPKM data is indicated by the red line scaled to the right y-axis. The plots are organized into three columns corresponding to data obtained from NRRL3357, AF13, Tox4, respectively, with each plot containing data from the 0, 10, and 20/25 mM H_2_O_2_ treatments in increasing order.

For kojic acid biosynthetic genes (Terabayashi et al., 2010), the *kojA* and *kojR/T* genes were constitutively expressed in all the isolates and did not vary significantly in expression in response to increasing stress (Figure 3C). The synaptic vesicle transporter SVOP, which is also involved in the biosynthesis of kojic acid (Terabayashi et al., 2010), was found to be differentially expressed in the examined isolates. In NRRL3357, the *SVOP* gene was upregulated by a log_2_(fold change) of 3.63 when comparing 0 and 10 mM H_2_O_2_, but was not significantly different between 0 and 20 mM (Dataset S3). For AF13, the *SVOP* gene was also upregulated by a log_2_(fold change) of 2.47 when comparing 0 and 25 mM H_2_O_2_, but was not significantly different between 0 and 10 mM H_2_O_2_ (Dataset S4). For Tox4, the *SVOP* gene was not significantly different between the control and the treatments, but was found to be downregulated when comparing 10 and 25 mM H_2_O_2_ indicating a slight reduction in response to higher levels of stress (Dataset S5). The expression of *kojA* and *SVOP* along with select aflatoxin and aflatrem genes were further confirmed with qPCR which were generally correlated with the FPKM data obtained by RNA sequencing (Figure 4).

### Stress responsive and related biochemical pathways regulated by increasing stress

**Figure 5:**
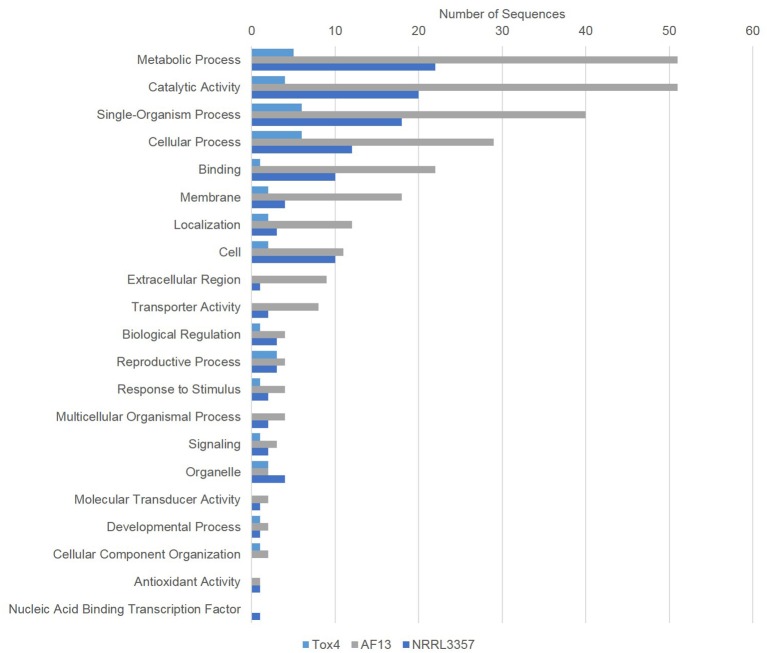
**Gene ontology (GO) analysis of genes differentially expressed between 0 and 20/25 mM H_2_O_2_**. Gene ontology (GO) analysis of significant DEGs between 0 and 20/25 mM H_2_O_2_ were determined using Blast2GO. Bars indicate the number of genes differentially expressed within each gene ontology group (biological process). The light blue bar represents Tox4, the gray bar AF13, and the dark blue bar NRRL3357.

In addition to the production mechanisms regulating secondary metabolite biosynthesis, additional stress responsive and associated biochemical pathway genes were also regulated in the isolates in response to increasing stress (Figure 5). Among the stress responsive genes, antioxidant and oxidase enzyme-encoding genes were among the most commonly regulated. In the NRRL3357 and AF13, thioredoxin peroxidase and thioredoxin reductase along with several cytochrome p450 monooxygenase genes were upregulated in response to increasing levels of oxidative stress (Datasets S3, S4). Interestingly, in Tox4 there were no antioxidant or oxidase genes regulated by 10 mM H_2_O_2_, and only one monooxygenase gene was regulated at 20 mM (Dataset S5).

In addition to these antioxidant and oxidase genes, genes related to fungal catabolism and antibiosis tolerance were also regulated by stress, particularly in AF13. Comparing 0 and 10 mM H_2_O_2_, two multiple drug resistance protein-encoding genes were upregulated and expressed at a high level (FPKM's of 1636.6 and 7697.7, respectively; Dataset S4). These genes were further upregulated when comparing 0 and 25 mM H_2_O_2_ (FPKMs of 2627.0 and 15,066.5, respectively), along with a third expressed at a lower level (Dataset S4). When comparing 0 and 25 mM H_2_O_2_, AF13 also exhibited the downregulation of a number of catabolic enzyme-encoding genes including acetyl xylan esterase, alpha-1,3-glucanase, aspergillopepsin 2 and F, class III and V chitinases, chitosanase, and penicillolysin/deuterolysin (Dataset S4). NRRL3357 also exhibited a similar downregulation in the chitin catabolic genes encoding beta-N-acetylhexosaminidase (nagA) and beta-N-hexosaminidase when comparing 0 and 20 mM H_2_O_2_ (Dataset S3). Iron metabolic genes were also regulated in NRRL3357 and AF13 in response to increasing stress. In NRRL3357, the siderophore biosynthesis acetylase gene *aceI* was upregulated when comparing 0 and 20 mM H_2_O_2_, and in AF13, a putative transferrin receptor gene was downregulated when comparing 0 and 25 mM H_2_O_2_ (Datasets S3, S4). NRRL3357 also exhibited an upregulation in cinnamoyl-CoA reductase gene expression when comparing 0 and 20 mM H_2_O_2_ (Dataset S3). Tox4 exhibited regulation of two phosphate signaling genes in response to increasing stress, but none of the aforementioned genes (Dataset S5).

### Fungal development-related genes are regulated by oxidative stress

Genes involved in the development of *A. flavus* and other *Aspergillus* spp. were also differentially expressed among the isolates. In NRRL3357, the gene encoding the Cis_2_His_2_ (C_2_H_2_) transcription factor flbC was upregulated along with a conidiation-specific family protein gene and a duf221 domain protein gene (Dataset S3). AF13 also exhibited an upregulation of a conidial hydrophobin gene in response to increasing stress (Dataset S4). In addition, genes involved in benzoate degradation were also regulated in response to stress in the isolates. NRRL3357 and AF13 both exhibited the upregulation of genes involved in benzoate degradation including a multiple inositol polyphosphate phosphatase and a benzoate 4-monooxygenase (Datasets S3, S4). Conversely, Tox4 exhibited a downregulation of an amidohydrolase family protein gene and a multiple inositol polyphosphate phosphatase gene (Dataset 5).

### bZIP transcription factors and promoter occurrence among differentially expressed genes

The orthologs of two bZIP transcription factor genes, *atfA* and *atf21*, were previously shown to regulate the expression of genes involved in both the biosynthesis of aflatoxin and sterigmatocystin, and oxidative stress responsive mechanisms (Roze et al., 2011). Here, *atf21* and *atfA* exhibited marginal increases in expression in NRRL3357 and AF13 under high levels of H_2_O_2_ stress (Figure S2). The ortholog of atf21 in *A. parasiticus*, atfB, has been well characterized and has been shown to bind a conserved promoter motif, AGCC(G/C)T(G/C)(A/G), upstream of several aflatoxin biosynthetic genes (Roze et al., 2011). Examination of the 1kb upstream regions of the significant DEGs observed in NRRL3357 showed that nine genes possess this predicted atf21 binding domain including a putative cytochrome p450 monooxygenase gene, *aflC*, and the siderophore biosynthesis acetylase *aceI* (S2).

### Gene co-expression is correlated with mycelial dry weight under oxidative stress

**Figure 6:**
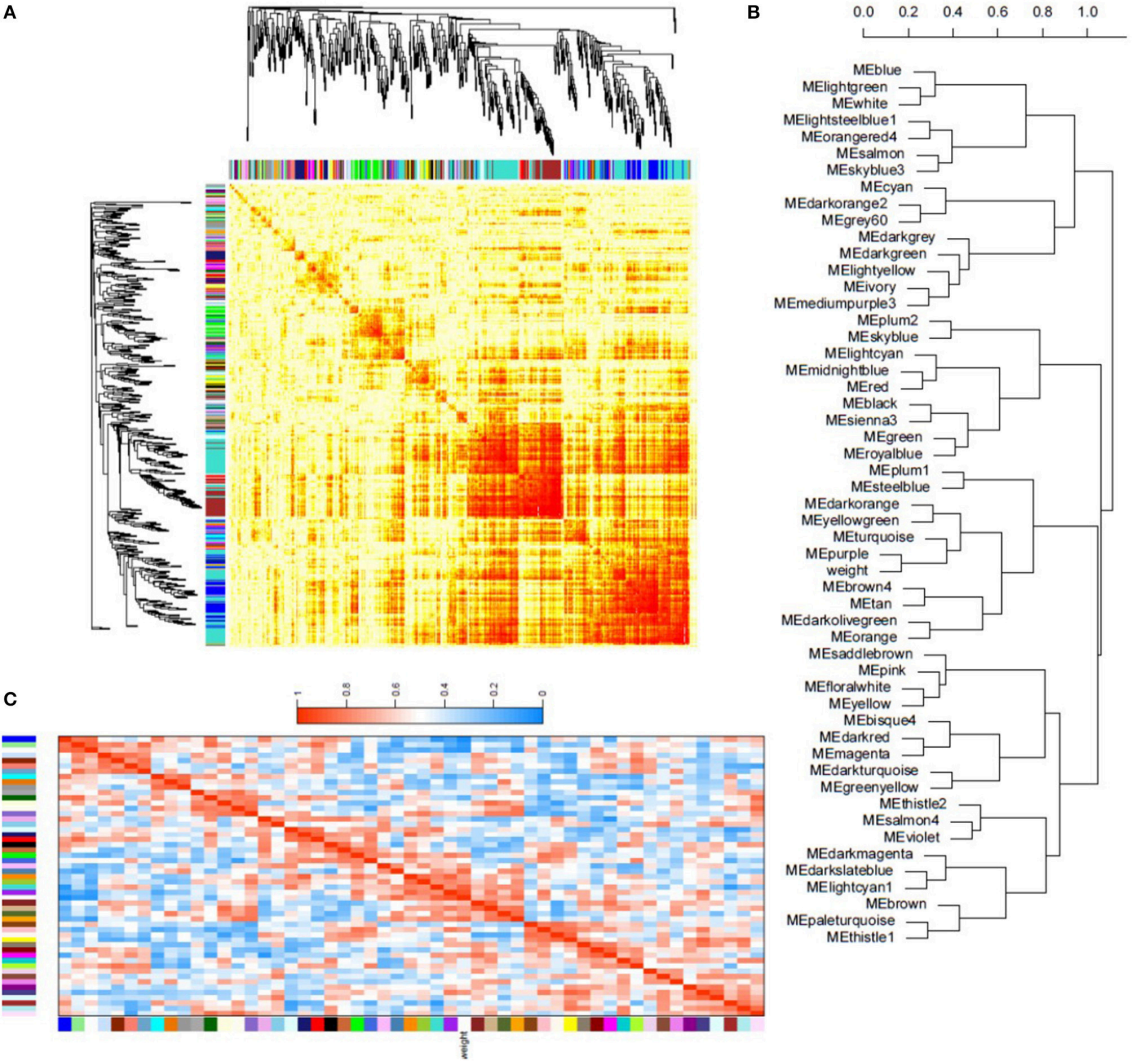
**Weighted gene co-expression network analysis of isolate transcriptional responses to oxidative stress. (A)** Topographical overlap matrix (TOM) of the co-expression network of 500 randomly selected genes in the dataset. Genes are sorted by hierarchical clustering, and represented by the rows and columns with the colors showing the strength of the associations between genes increasing from white to red. Genes clustered tightly together are assigned to color-coded modules shown below the dendrograms. **(B)** Dendrogram produced by hierarchical clustering of the eigengene network showing the relationship of the modules and isolate dry weight under oxidative stress. **(C)** Eigengene adjacency heatmap showing the correlation between the modules and isolate dry weight. Mutual correlations between corresponding modules is higher than most module associations with dry weight, however the purple and turquoise modules exhibit a strong positive correlation with dry weight. In contrast, the blue module shows a strong negative correlation. Eigengene adjacency, which reflects correlation in the heatmap, increases from blue to red in the heatmap.

In order to identify groups of genes whose expression is directly correlated with isolate oxidative stress tolerance, a co-expression analysis was performed for all isolates and treatments and correlated with previously obtained fungal dry weights (Figure 6). Co-expressed genes were grouped based on expression profile similarities into color-coded modules, which were further separated by hierarchical clustering. Gene network analysis revealed that the largest modules in terms of gene content were the turquoise, blue, and brown modules (Figure 6A).

Module-trait association analysis was then performed to correlate fungal dry weights under each treatment. The blue, purple, and turquoise modules were found to be associated with isolate dry weight under oxidative stress (Figure 6B). Statistical eigengene expression and fungal dry weight were also found to be significantly correlated with the purple (*r* = 0.83, *p* = 0.006) and turquoise (*r* = 0.73, *p* = 0.02) module genes showing a strong positive correlation with isolate dry weight and the blue module genes showing a strong negative correlation (*r* = −0.93, *p* = 0.0002) with isolate dry weight (Figure 6C). Gene ontology (GO) enrichment analysis of these modules showed that the blue module was enriched for simple sugar metabolism terms, the purple module for regulation of gene expression and metabolic processes, and the turquoise module for translational mechanisms and additional metabolic processes (Dataset S6). The blue and turquoise modules were also found to contain the aflatoxin biosynthetic genes while the purple module contained several MFS transporter and antioxidant genes (Dataset S6).

## Discussion

The production of secondary metabolites in soil dwelling fungi has been shown to be regulated by a number of factors, particularly environmental stress (Calvo and Cary, 2015). Aflatoxin production by *A. flavus* has been shown to be stimulated by a number of compounds *in vitro*, with reactive oxygen species (ROS) being of particular interest given their prevalence in host plant defense signaling and environmental stress, particularly drought stress, responses (Jayashree and Subramanyam, 2000; Bhattacharjee, 2005; Roze et al., 2013; Yang et al., 2015, 2016). Such ROS have also been shown to be required for the production of aflatoxin with treatment with antioxidant compounds resulting in reduced aflatoxin production and fungal growth (Nesci et al., 2003; Passone et al., 2005; Mahoney et al., 2010). However, the practical role of aflatoxin production in *A. flavus* biology is still not well understood. In order to better understand to biological role of aflatoxin production in *A. flavus*, and its role in oxidative stress responses, we examined the transcriptomes of three aflatoxigenic field isolates exposed to increasing levels of H_2_O_2_-derived oxidative stress.

The isolates which had previously exhibited greater levels of H_2_O_2_ tolerance and aflatoxin production (Fountain et al., 2015), Tox4 and AF13, exhibited a fewer numbers of significantly differentially expressed genes in comparison to the isolate with less tolerance, NRRL3357 (Table 1). This trend also resulted in strong correlations between previously observed stress tolerance and the numbers of DEGs (Figure S1). Interestingly, the AF13 isolate exhibits both a similar number and composition of DEGs at 25 mM H_2_O_2_ in comparison to NRRL3357 at 10 mM (Figure S1; Datasets S3, S4). The requirement of additional stress to elicit a similar response is commensurate with the observed stress tolerance and aflatoxin production capabilities of AF13 and NRRL3357 (Fountain et al., 2015). Tox4, however, may possess stress responsive mechanisms which at more vigorously earlier accounting for the reduced DEG counts observed in this study. Together these results indicate, as has been previously suggested in the literature, that aflatoxin production may contribute to oxidative stress tolerance in *Aspergillus* spp. (Narasaiah et al., 2006; Roze et al., 2013, 2015; Fountain et al., 2014, 2016).

Examining the expression of aflatoxin biosynthetic genes showed that the NRRL3357 isolate exhibited the up-regulation and expression of a greater number of these genes than the other isolates examined in the study (Table 1, Figure 3A). An increase in expression, though to a lesser extent, was also observed for AF13 under higher levels of stress, but not for Tox4 (Figure 3A). This was surprising given that aflatoxin biosynthetic gene expression is typically higher with periods in fungal development *in vitro* that coincide with the highest levels of aflatoxin production, approximately 2 to 6 days in conducive media (Davis et al., 1966). The high level of expression of aflatoxin genes in NRRL3357 at 7 days under high levels of stress coupled with the reduced fungal biomass and conidiation observed for this isolate and not with AF13 or Tox4 in our preliminary study (Fountain et al., 2015), suggests that a delay in fungal growth and development may contribute to prolonged aflatoxin gene expression in addition to stimulation provided by oxidative stress. In addition, these differential expression patterns of aflatoxin biosynthetic genes may also be indicative of post-transcriptional regulation of aflatoxin production and oxidative stress responses at either the RNA or protein level. For example, there has been a low degree of correlation observed between gene expression and proteomics data for traits such as temperature stress (*r* = 0.14) suggesting post-transcriptional regulation of protein accumulation in *A. flavus* (Bai et al., 2015). Also, given that aflatoxin biosynthetic gene expression can be detected as early as 8 h in culture, there is ample opportunity for such regulation to have a significant effect on later responses to stress and warrants further investigation (Price et al., 2006).

However, factors in addition to aflatoxin production have been shown to be at play which influence *A. flavus* tolerance to oxidative stress. For example, Reverberi et al. (2012) examined the effects of excessive peroxisome proliferation in a mutant isolate expressing the P33 gene. They found that the P33-containing isolate exhibited elevated ROS and oxylipin accumulation commensurate with excess peroxisomes, and countered this with increased aflatoxin production and higher antioxidant enzyme activity compared to the wildtype isolate. The mutant isolate also exhibited similar fungal growth but reduced conidiation compared to the wildtype. Elevated expression of antioxidant and aflatoxin genes at the time point examined in our study would imply that continued stress is still being experienced by NRRL3357, and not to the same extent in AF13 or Tox4. This suggests that NRRL3357 is less able to sequester ROS and remediate its damage than the other isolates. Our previous observation that culturing these isolates in an aflatoxin production non-conducive medium results only in a slight reduction in stress tolerance also suggests that additional oxidative stress remediation mechanisms are at play in all isolates in addition to aflatoxin production (Fountain et al., 2015, 2016).

Two bZIP transcription factor genes, *atf21* and *atfA*, whose homologs in other *Aspergillus* spp. have been shown to integrate oxidative stress responses and sterigmatocystin/aflatoxin biosynthesis regulation (Sakamoto et al., 2008, 2009; Lara-Rojas et al., 2011; Roze et al., 2011, 2013; Hong et al., 2013a,b), also displayed some marginally significant increases in expression in NRRL3357 and AF13 (Figure S2). Atf21, whose ortholog in *A. parasiticus*, atfB, has been well characterized (Roze et al., 2011), was found to bind a conserved motif in the promoter regions of several aflatoxin biosynthetic genes. This same motif was found in the upstream promoter regions of nine DEGs in NRRL3357 under high levels of stress, and include aflatoxin production, oxidative stress response, and siderophore biosynthetic genes (Figure S2). This suggests that these transcription factors may actively regulate gene expression following aflatoxin production initiation (Roze et al., 2011, 2013) and further supports the case for their involvement in stress tolerance. Also, the co-expression analysis identified several modules of genes exhibiting similar expression profiles which were closely associated with fungal biomass under oxidative stress, including the blue, purple, and turquoise modules (Figure 6C). These modules contain genes enriched for carbon metabolism and gene expression regulation, but also contain antioxidant enzyme-encoding genes and the aflatoxin biosynthetic genes (Dataset S6). This further supports the observed association of aflatoxin production and oxidative stress tolerance in *A. flavus*.

The link between aflatoxin production and fungal development has been widely documented in the literature to involve G-protein mediated signaling along with numerous transcription factors and regulatory proteins (Amaike and Keller, 2011). Here, velvetA (*VeA*), a key developmental regulator influencing sclerotial and conidia formation in response to light levels (Bayram et al., 2008), was constitutively expressed regardless of stress levels while conidiation-related genes such as *flbC*, a conidiation-specific family protein gene, and a duf221 domain protein gene were also up-regulated under stress conditions (Ben-Ami et al., 2010; Kwon et al., 2010). This suggests as has been previously observed (Fountain et al., 2015; Roze et al., 2015) that oxidative stress can stimulate conidiation in *Aspergillus* spp. Benzoate and amino benzoate degradation genes were also regulated in response to stress (Datasets S3–S5). These compounds have been shown to reduce aflatoxin production and slow fungal growth and development when applied *in vitro* providing further indication that fungal development may be affected by oxidative stress exposure (Chipley and Uraih, 1980).

Chitin catabolism-related genes tended to be down-regulated under oxidative stress (Datasets S3, S4) which may reduce cell wall degradation and enhance cell well integrity to protect against oxidative stress (Fuchs and Mylonakis, 2009; Jain et al., 2011; Fountain et al., 2016). Also, antibiosis-related genes encoding multiple drug resistance proteins were also upregulated, particularly in AF13, in response to stress. This may provide additional protection for this isolate in competition with other soilborne or plant parasitic microorganisms, especially under environmental stress (Daguerre et al., 2014). NRRL3357 also exhibited an upregulation in cinnamoyl-CoA reductase gene expression in response to stress (Dataset S3). This is noteworthy since it was only fairly recently found that phenylpropanoid metabolic genes were present in the genomes of fungi including the Aspergilli (Seshime et al., 2005). Several members of this class of compounds have been found to influence aflatoxin production by *A. flavus in vitro*, and have been shown to function as antioxidants which could enhance stress tolerance and competitive capability of the isolates (Chipley and Uraih, 1980). This may indicate that phenylpropanoids or flavonoids produced in host plants along with ROS during drought stress may also be able to influence the production of aflatoxin by *A. flavus* and warrants further study (Alvarez et al., 2008; Payton et al., 2009). Genes encoding antioxidant enzymes such as thioredoxin peroxidase and thioredoxin reductase were also significantly regulated in response to increasing stress. In the co-expression analysis, the purple module, which exhibited the highest positive correlation with fungal biomass, also contained antioxidant enzyme encoding genes such as glutathione-S-transferase (Dataset S6).

Two additional secondary metabolite biosynthetic gene clusters were also induced by oxidative stress, aflatrem and kojic acid. Aflatrem is a tremerogenic mycotoxin which causes stagger's syndrome in cattle and neurological effects in mammals (Nicholson et al., 2009). Aflatrem is also proposed to function in fungivory prevention for soilborne fungi (TePaske and Gloer, 1992). The biosynthesis of aflatrem is carried out by the indole-diterpenoid (isoprenoid) pathway in *Aspergillus* spp. and shares with aflatoxin biosynthesis the commonality of cytochrome p450 monooxygenase activity (Nicholson et al., 2009; Calvo and Cary, 2015). This along with the activities of various oxidases has led to the proposition that aflatoxin production may function in the alleviation of oxidative stress by promoting antioxidant enzyme production and conidial oxidative stress tolerance through secondary ROS production (Roze et al., 2015; Fountain et al., 2016). We also propose that this process may result in the fixation of excess molecular oxygen (O_2_) and detoxified ROS into the aflatoxin molecules which are then secreted from the cell as an indirect means of relieving oxidative stress (Fountain et al., 2016). Alone or in combination, these two proposals would also suggest that isolates which produce higher levels of aflatoxin would exhibit greater oxidative stress tolerance, a trend observed here and in our preliminary study, but which will require further study to confirm (Fountain et al., 2015, 2016). Given the prevalence of cytochrome p450 monooxygenase activity in the aflatrem biosynthetic mechanism, it is possible that a similar phenomenon may occur which is also in need of further investigation.

Kojic acid is a bi- or tri-dentate iron chelating antioxidant compound produced by several species of Aspergilli including *A. sojae* (Chang et al., 2010). The compound and derivatives of it are useful for the treatment of iron overload in people with thalassemia who accumulate dangerously high levels of iron in their blood stream following multiple transfusions (Nurchi et al., 2016). The iron chelating properties of kojic acid may also, as in the case of cyclopiazonic acid (CPA), may help soil-borne fungi in the remediation of iron starvation under stress conditions by fixing additional iron cations and outcompeting other microbes (Chang et al., 2009). This may also be affected by oxidative stress in *A. flavus* since siderophore biosynthetic genes were also found to be regulated in AF13 and NRRL3357. Also, the chelation of excess iron cations, particularly Fe^2+^, may prevent Fenton reaction-derived ROS such as hydroxyl radicals (OH^.−^) from forming and resulting in additional cellular damage (Fenton, 1894). Therefore, the production of kojic acid may also supplement *A. flavus* oxidative stress tolerance. Also, these same hydroxyl radicals have also been shown to influence aflatoxin production with treatment of *A. flavus* mycelia with DMSO, a hydroxyl radical scavenger, resulting in significantly reduced aflatoxin production and sclerotial differentiation (Grintzalis et al., 2014).

Together, these results provide an insight into a potentially coordinated secondary metabolite response to oxidative stress in *A. flavus*. While the biological function of the aflatoxin compound in fungal biology remains unclear, it seems possible that the aflatoxin production process may contribute to oxidative stress tolerance. However, in the absence of aflatoxin production capability, the production of other secondary metabolites may compensate for any reduction in stress tolerance, and may be of interest in the biology of atoxigenic biological control isolates. This role of oxidative stress in stimulating aflatoxin production in *A. flavus* is made all the more interesting by the observed accumulation of ROS in drought stressed host plant tissues (Yang et al., 2015, 2016), and the correlation of elevated antioxidant enzyme and compound accumulation in hosts resistant to aflatoxin contamination (Chitarrini et al., 2014). Together, this may be a contributing factor to the observed correlation between drought stress susceptibility and aflatoxin contamination in host plant species such as maize and peanut (Holbrook et al., 2009; Kebede et al., 2012). The role of oxidative stress in both secondary metabolite production and fungal primary metabolism may also provide a basis for identifying host defense attributes which contribute to reduced aflatoxin contamination in the form of selectable biomarkers and in the selection and characterization of novel resistance genes and genetic markers identified through quantitative trait loci (QTL) studies for marker assisted selection (MAS) and cultivar resistance improvement.

## Author contributions

JF: Performed the experiments, assisted in data analysis, and wrote the manuscript; PB: Performed the data analysis; MP and SN: Assisted with manuscript preparation and data analysis; VK: Performed the sequencing; AJ and AC: Assisted in RNA preparation for sequencing. LY, RL, and RK: Contributed to the design and project discussion; RV and BG: Conceived and supervised the project, secured funding, and revised and submitted the manuscript.

### Conflict of interest statement

The authors declare that the research was conducted in the absence of any commercial or financial relationships that could be construed as a potential conflict of interest.

## Supplementary material

Figure S1 **Linear regression analysis of the numbers of differentially expressed genes relative to isolate H_2_O_2_ tolerance**. The numbers of significantly, differentially expressed genes were plotted relative to the observed maximum H_2_O_2_ level tolerated by the isolates observed in Fountain et al. (2015). Significant correlations were observed when comparing 0 and 10 mM H_2_O_2_ (*R*^2^ = 0.9580), and 10 and 20/2 5 mM H_2_O_2_ (*R*^2^ = 0.9174). The correlation between 0 and 20/25 mM H_2_O_2_ was not as significant (*R*^2^ = 0.4332) possibly due to a similarity between NRRL3357 and AF13 responses to high levels of stress. Numerical data can be found in Table 1.

Figure S2 **FPKMs of genes encoding the bZIP transcription factors atf21 and aftA**. The expression of the bZIP transcription factor genes *aft21* **(A)** and *aftA* **(B)** was marginally, significantly regulated in response to high levels of stress in NRRL3357 and AF13. While in the study, a *p*-value and a *q*-value ≤ 0.05 with a log_2_(fold change) ≥ 2 were considered to be significant, these genes were numerically and marginally expressed higher in response to stress. Green bars in the plot represent 0 mM H_2_O_2_, blue bars 10 mM H_2_O_2_, and yellow bars 20/25 mM H_2_O_2_. The bracket displays the *p*- and a *q*-values observed between the 0 and 20/25 mM H_2_O_2_ treatments. Promoter analysis with MEME identified nine genes **(C)** in NRRL3357 which were differentially expressed between 0 and 20 mM H_2_O_2_ that contain the conserved motif **(D)** recognized by the atf21 ortholog, atfB, in *A. parasiticus* (Roze et al., 2011).

Table S1 **Primer sequences used in qPCR Analyses**.

Table S2 **Principal component analysis (PCA) Eigenvalues and variances**.

Dataset S1 **Gene expression FPKM data for all isolates and treatments**.

Dataset S2 **Genes expressed with FPKM ≥ 2 exclusively in each isolate**.

Dataset S3 **Significantly differentially expressed genes in the NRRL3357 isolate in response to H_2_O_2_ stress**.

Dataset S4 **Significantly differentially expressed genes in the AF13 isolate in response to H_2_O_2_ stress**.

Dataset S5 **Significantly differentially expressed genes in the Tox4 isolate in response to H_2_O_2_ stress**.

Dataset S6 **Weighted gene co-expression analysis**.

### Additional information

**Data availability:** Raw and processed RNA sequencing data can be accessed at the National Center for Biotechnology Information (NCBI) Sequence Read Archive (SRA) with accession number SRP094082 under BioProject PRJNA348383.

## References

[B1] Alvarez S. Marsh E. L. Schroeder S. G. Schachtman D. P. (2008). Metabolomic and proteomic changes in the xylem sap of maize under drought. Plant Cell Environ. 31, 325–340. 10.1111/j.1365-3040.2007.01770.x 18088330

[B2] Amaike S. Keller N. P. (2011). Aspergillus flavus. Ann. Rev. Phytopathol. 49, 107–133. 10.1146/annurev-phyto-072910-095221 21513456

[B3] Andrade P. D. Caldas E. D. (2015). Aflatoxins in cereals: worldwide occurrence and dietary risk assessment. World Mycotoxin J. 8, 415–431. 10.3920/WMJ2014.1847

[B4] Bai Y. Wang S. Zhong H. Yang Q. Zhang F. Zhuang Z.. (2015). Integrative analyses reveal transcriptome-proteome correlation in biological pathways and secondary metabolism clusters in A. flavus in response to temperature. Sci. Rep. 5:15482. 10.1038/srep14582 26416011 PMC4586720

[B5] Baidya S. Duran R. M. Lohmar J. M. Harris-Coward P. Y. Cary J. W. Hong S. Y. (2014). VeA is associated with the response to oxidative stress in the aflatoxin producer *Aspergillus flavus*. Eukaryotic Cell. 13, 1095–1103. 10.1128/EC.00099-14 24951443 PMC4135802

[B6] Bailey T. L. Boden M. Buske F. A. Frith M. Grant C. E. Clementi L. (2009). MEME Suite: tools for motif discovery and searching. Nuclic Acids Res. 35, 202–208. 10.1093/nar/gkp335 PMC2703892 19458158

[B7] Bayram O. Krappmann S. Ni M. Bok J. W. Helmstaedt K. Valerius O. (2008). VelB/VeA/LaeA complex coordinates light signal with fungal development and secondary metabolism. Science 320, 1504–1506. 10.1126/science.1155888 18556559

[B8] Ben-Ami R. Varga J. Lewis R. E. May G. S. Nierman W. C. Kontoyiannis D. P. (2010). Characterization of a 5-azacytidine-induced developmental *Aspergillus fumigatus* variant. Virulence 1, 164–173. 10.4161/viru.1.3.11750 21178435 PMC3265789

[B9] Bhattacharjee S. (2005). Reactive oxygen species and oxidative burst: roles in stress, senescence and signal. Curr. Sci. India. 89, 1113–1121.

[B10] Calvo A. M. Cary J. W. (2015). Association of fungal secondary metabolism and sclerotial biology. Front. Microbiol. 6:62. 10.3389/fmicb.2015.00062 25762985 PMC4329819

[B11] Chanda A. Roze L. V. Kang S. Artymovich K. A. Hicks G. R. Raikhel N. V.. (2009). A key role for vesicles in fungal secondary metabolism. Proc. Nat. Acad. Sci. U.S.A. 106, 19533–19538. 10.1073/pnas.0907416106 19889978 PMC2773199

[B12] Chanda A. Roze L. V. Linz J. E. (2010). A possible role for exocytosis in aflatoxin export in *Aspergillus parasiticus*. Eukaryotic Cell. 9, 1724–1727. 10.1128/EC.00118-10 20870882 PMC2976301

[B13] Chang P. K. Ehrlich K. C. Fujii I. (2009). Cyclopiazonic acid biosynthesis of *Aspergillus flavus* and *Aspergillus oryzae*. Toxins 1, 74–99. 10.3390/toxins1020074 22069533 PMC3202784

[B14] Chang P. K. Scharfenstein L. L. Luo M. Mahoney N. Molyneux R. J. Yu J.. (2010). Loss of msnA, a putative stress regulatory gene, in *Aspergillus parasiticus* and *Aspergillus flavus* increased production of conidia, aflatoxins and kojic acid. Toxins 3, 82–104. 10.3390/toxins3010082 22069691 PMC3210457

[B15] Chipley J. R. Uraih N. (1980). Inhibition of Aspergillus growth and aflatoxin release by derivatives of benzoic acid. App. Environ. Microbiol. 40, 352–357. 6781406 10.1128/aem.40.2.352-357.1980 PMC291580

[B16] Chitarrini G. Nobili C. Pinzari F. Antonini A. De Rossi P. Del Fiore A.. (2014). Buckwheat achenes antioxidant profile modulates *Aspergillus flavus* growth and aflatoxin production. Int. J. Food Microbiol. 189, 1–10. 10.1016/j.ijfoodmicro.2014.07.029 25108759

[B17] Daguerre Y. Siegel K. Edel-Hermann V. Steinberg C. (2014). Fungal proteins and genes associated with biocontrol mechanisms of soil-borne pathogens: a review. Fungal Biol. Rev. 28, 97–125. 10.1016/j.fbr.2014.11.001

[B18] Davis N. D. Diener U. L. Eldridge D. W. (1966). Production of aflatoxins B1 and G1 by *Aspergillus flavus* in a semisynthetic medium. App. Microbiol. 14, 378–380. 5970823 10.1128/am.14.3.378-380.1966 PMC546720

[B19] Diener U. L. Asquith R. L. Dickens J. W. (1983). Aflatoxin and *Aspergillus flavus* in corn. So. Coop. Ser. Bull. 279:112.

[B20] Diener U. L. Cole R. J. Sanders T. H. Payne G. A. Lee L. S. Klich M. A. (1987). Epidemiology of aflatoxin formation by *Aspergillus flavus*. Annu. Rev. Phytopathol. 25, 249–270. 10.1146/annurev.py.25.090187.001341

[B21] Fenton H. J. H. (1894). Oxidation of tartaric acid in presence of iron. J. Chem. Soc. Trans. 65, 899–910. 10.1039/CT8946500899

[B22] Fountain J. C. Bajaj P. Pandey M. Nayak S. N. Yang L. Kumar V.. (2016). Oxidative stress and carbon metabolism influence *Aspergillus flavus* secondary metabolite production and transcriptome composition. Sci. Rep. 6:38747. 10.1038/srep38747 27941917 PMC5150527

[B23] Fountain J. C. Scully B. T. Chen Z. Y. Gold S. E. Glenn A. E. Abbas H. K.. (2015). Effects of hydrogen peroxide on different toxigenic and atoxigenic isolates of *Aspergillus flavus*. Toxins 7, 2985–2999. 10.3390/toxins7082985 26251922 PMC4549735

[B24] Fountain J. C. Scully B. T. Ni X. Kemerait R. C. Lee R. D. Chen Z. Y.. (2014). Environmental influences on maize-*Aspergillus flavus* interactions and aflatoxin production. Front. Microbiol. 5:40. 10.3389/fmicb.2014.00040 24550905 PMC3913990

[B25] Fuchs B. B. Mylonakis E. (2009). Our paths might cross: the role of the fungal cell wall integrity pathway in stress response and cross talk with other stress response pathways. Eukaryotic Cell 8, 1616–1625. 10.1128/EC.00193-09 19717745 PMC2772411

[B26] Gao X. Brodhagen M. Isakeit T. Brown S. H. Göbel C. Betran J.. (2009). Inactivation of the lipoxygenase ZmLOX3 increases susceptibility of maize to *Aspergillus* spp. Mol. Plant Mic. Interact. 22, 222–231. 10.1094/MPMI-22-2-0222 19132874 PMC4545248

[B27] Grintzalis K. Vernardis S. Klapa M. Georgiou C. D. (2014). Role of oxidative stress in sclerotial differentiation and aflatoxin B1 biosynthesis in *Aspergillus flavus*. App. Environ. Microbiol. 80, 5561–5571. 10.1128/AEM.01282-14 25002424 PMC4178614

[B28] Guo B. Chen Z. Y. Lee R. D. Scully B. T. (2008). Drought stress and preharvest aflatoxin contamination in agricultural commodity: genetics, genomics and proteomics. J. Int. Plant Biol. 50, 1281–1291. 10.1111/j.1744-7909.2008.00739.x 19017115

[B29] Guo B. Russin J. S. Cleveland T. E. Brown R. L. Damann K. E. (1996). Evidence for cutinase production by *Aspergillus flavus* and its possible role in infection of corn kernels. Phytopathology 86, 824–829. 10.1094/Phyto-86-824

[B30] Guo B. Yu J. Ni X. Lee R. D. Kemerait R. C. Scully B. T. (2012). Crop stress and aflatoxin contamination: perspectives and prevention strategies, in Crop Stress and its Management: Perspectives and Strategies, eds Venkateswarlu B. Shankerk A. K. Shanker C. Makeswari M. (New York, NY: Springer), 399–427.

[B31] Holbrook C. C. Guo B. Wilson D. M. Timper P. (2009). The US breeding program to develop peanut with drought tolerance and reduced aflatoxin contamination. Peanut Sci. 36, 50–53. 10.3146/AT07-009.1

[B32] Hong S. Y. Roze L. V. Linz J. E. (2013a). Oxidative stress-related transcription factors in the regulation of secondary metabolism. Toxins 5, 683–702. 10.3390/toxins5040683 23598564 PMC3705287

[B33] Hong S. Y. Roze L. V. Wee J. Linz J. E. (2013b). Evidence that a transcription factor regulatory network coordinates oxidative stress response and secondary metabolism in aspergilli. MicrobiologyOpen 2, 144–160. 10.1002/mbo3.63 23281343 PMC3584220

[B34] Jain R. Valiante V. Remme N. Docimo T. Heinekamp T. Hertweck C.. (2011). The MAP kinase MpkA controls cell wall integrity, oxidative stress response, gliotoxin production and iron adaptation in *Aspergillus fumigatus*. Mol. Microbiol. 82, 39–53. 10.1111/j.1365-2958.2011.07778.x 21883519 PMC3229709

[B35] Jayashree T. Subramanyam C. (2000). Oxidative stress as a prerequisite for aflatoxin production by *Aspergillus parasiticus*. Free Rad. Biol. Med. 29, 981–985. 10.1016/S0891-5849(00)00398-1 11084286

[B36] Jiang T. Fountain J. C. Davis G. Kemerait R. C. Scully B. T. Lee R. D. (2012). Root morphology and gene expression analysis in response to drought stress in maize (*Zea mays*). Plant Mol. Biol. Rep. 30, 360–369. 10.1007/s11105-011-0347-9

[B37] Kebede H. Abbas H. K. Fisher D. K. Bellaloui N. (2012). Relationship between aflatoxin contamination and physiological responses of corn plants under drought and heat stress. Toxins 4, 1385–1403. 10.3390/toxins4111385 23202322 PMC3509714

[B38] Kew M. C. (2013). Aflatoxins as a cause of hepatocellular carcinoma. J. Gastrointestin. Liver Dis. 22, 305–310. 24078988

[B39] Kwon N. J. Garzia A. Espeso E. A. Ugalde U. Yu J. H. (2010). FlbC is a putative nuclear C2H_2_ transcription factor regulating development in *Aspergillus nidulans*. Mol. Microbiol. 77, 1203–1219. 10.1111/j.1365-2958.2010.07282.x 20624219

[B40] Langfelder P. Horvath S. (2008). WGCNA: an R package for weighted correlation network analysis. BMC Bioinform. 9:559. 10.1186/1471-2105-9-559 19114008 PMC2631488

[B41] Lara-Rojas F. Sànchez O. Kawasaki L. Aguirre J. (2011). *Aspergillus nidulans* transcription factor AtfA interacts with the MAPK SakA to regulate general stress responses, development and spore functions. Mol. Microbiol. 80, 436–454. 10.1111/j.1365-2958.2011.07581.x 21320182 PMC3108070

[B42] Livak K. J. Schmittgen T. D. (2001). Analysis of relative gene expression data using real-time quantitative PCR and the 2^−ΔΔCt^ method. Methods 25, 402–408. 10.1006/meth.2001.1262 11846609

[B43] Mahoney N. Molyneux R. Kim J. Campbell B. Waiss A. Hagerman A. (2010). Aflatoxigenesis induced in *Aspergillus flavus* by oxidative stress and reduction by phenolic antioxidants from tree nuts. World Mycotoxin J. 3, 49–57. 10.3920/WMJ2009.1185

[B44] Matumba L. Van Poucke C. Monjerezi M. Ediage E. N. De Saeger S. (2015). Concentrating aflatoxins on the domestic market through groundnut export: a focus on Malawian groundnut value and supply chain. Food Control. 51, 236–239. 10.1016/j.foodcont.2014.11.035

[B45] Narasaiah K. V. Sashidhar R. B. Subramanyam C. (2006). Biochemical analysis of oxidative stress in the production of aflatoxin and its precursor intermediates. Mycopathologia 162, 179–189. 10.1007/s11046-006-0052-7 16944285

[B46] Nesci A. Rodriguez M. Etcheverry M. (2003). Control of Aspergillus growth and aflatoxin production using antioxidants at different conditions of water activity and pH. J. App. Microbiol. 95, 279–287. 10.1046/j.1365-2672.2003.01973.x 12859759

[B47] Nicholson M. J. Koulman A. Monahan B. J. Pritchard B. L. Payne G. A. Scott B. (2009). Identification of two aflatrem biosynthesis gene loci in *Aspergillus flavus* and metabolic engineering of *Penicillium paxilli* to elucidate their function. Appl. Environ. Microbiol. 75, 7469–7481. 10.1128/AEM.02146-08 19801473 PMC2786402

[B48] Nurchi V. M. Crisponi G. Lachowicz J. I. Medici S. Peana M. Zoroddu M. A. (2016). Chemical features of in use and in progress chelators for iron overload. J. Trace Elem. Med. Biol. 38, 10–18. 10.1016/j.jtemb.2016.05.010 27365273

[B49] Pandey M. K. Monyo E. Ozias-Akins P. Liang X. Guimarães P. Nigam S. N.. (2012). Advances in Arachis genomics for peanut improvement. Biotech. Adv. 30, 639–651. 10.1016/j.biotechadv.2011.11.001 22094114

[B50] Park H. S. Ni M. Jeong K. C. Kim Y. H. Yu J. H. (2012). The role, interaction and regulation of the Velvet regulator VelB in *Aspergillus nidulans*. PLoS ONE 7:e45935. 10.1371/journal.pone.0045935 23049895 PMC3457981

[B51] Passone M. A. Resnik S. L. Etcheverry M. G. (2005). *In vitro* effect of phenolic antioxidants on germination, growth and aflatoxin B1 accumulation by peanut Aspergillus section Flavi. J. App. Microbiol. 99, 682–691. 10.1111/j.1365-2672.2005.02661.x 16108810

[B52] Payton P. Kottapalli K. R. Rowland D. Faircloth W. Guo B. Burow M.. (2009). Gene expression profiling in peanut using high density oligonucleotide microarrays. BMC Genomics 10:265. 10.1186/1471-2164-10-265 19523230 PMC2703657

[B53] Price M. S. Yu J. Nierman W. C. Kim H. S. Pritchard B. Jacobus C. A.. (2006). The aflatoxin pathway regulator AflR induces gene transcription inside and outside of the aflatoxin biosynthetic cluster. FEMS Microbiol. Lett. 255, 275–279. 10.1111/j.1574-6968.2005.00084.x 16448506

[B54] Reverberi M. Punelli M. Smith C. A. Zjalic S. Scarpari M. Scala V.. (2012). How peroxisomes affect aflatoxin biosynthesis in *Aspergillus flavus*. PLoS ONE 7:e48097. 10.1371/journal.pone.0048097 23094106 PMC3477134

[B55] Reverberi M. Ricelli A. Zjalic S. Fabbri A. A. Fanelli C. (2010). Natural functions of mycotoxins and control of their biosynthesis in fungi. Appl. Microbiol. Biotech. 87, 899–911. 10.1007/s00253-010-2657-5 20495914

[B56] Reverberi M. Zjalic S. Ricelli A. Punelli F. Camera E. Fabbri C.. (2008). Modulation of antioxidant defense in *Aspergillus parasiticus* is involved in aflatoxin biosynthesis: a role for the ApyapA gene. Eukaryotic Cell 7, 988–1000. 10.1128/EC.00228-07 18441122 PMC2446656

[B57] Roze L. V. Chanda A. Wee J. Awad D. Linz J. E. (2011). Stress-related transcription factor AtfB integrates secondary metabolism with oxidative stress response in Aspergilli. J. Biol. Chem. 286, 35137–35148. 10.1074/jbc.M111.253468 21808056 PMC3186425

[B58] Roze L. V. Hong S. Y. Linz J. E. (2013). Aflatoxin biosynthesis: current frontiers. Annu. Rev. Food Sci. Tech. 4, 293–311. 10.1146/annurev-food-083012-123702 23244396

[B59] Roze L. V. Laivenieks M. Hong S. Y. Wee J. Wong S. S. Vanos B.. (2015). Aflatoxin biosynthesis is a novel source of reactive oxygen species – A potential redox signal to initiate resistance to oxidative stress? Toxins 7, 1411–1430. 10.3390/toxins7051411 25928133 PMC4448155

[B60] Sakamoto K. Arima T. Iwashita K. Yamada O. Gomi Y. Akita O. (2008). *Aspergillus oryzae* atfB encodes a transcription factor required for stress tolerance in conidia. Fungal Genet. Biol. 45, 922–932. 10.1016/j.fgb.2008.03.009 18448366

[B61] Sakamoto K. Iwashita K. Yamada O. Kobayashi K. Mizuno A. Akita O.. (2009). *Aspergillus oryzae* atfA controls conidial germination and stress tolerance. Fungal Genet. Biol. 46, 887–897. 10.1016/j.fgb.2009.09.004 19770065

[B62] Seshime Y. Juvvadi P. R. Fujii I. Kitamoto K. (2005). Genomic evidences for the existence of a phenylpropanoid metabolic pathway in *Aspergillus oryzae*. Biochem. Biophys. Res. Comm. 337, 747–751. 10.1016/j.bbrc.2005.08.233 16182237

[B63] Stajich J. E. Harris T. Brunk B. P. Brestelli J. Fischer S. Harb O. S.. (2012). FungiDB: an integrated functional genomics database for fungi. Nuc. Acids Res. 40, 675–681. 10.1093/nar/gkr918 22064857 PMC3245123

[B64] TePaske M. R. Gloer J. B. (1992). Aflavarin and B-aflatrem: New anti-insectan metabolites from the sclerotia of *Aspergillus flavus*. J. Nat. Prod. 55, 1080–1086. 10.1021/np50086a008

[B65] Terabayashi Y. Sano M. Yamane N. Marui J. Tamano K. Sagara J.. (2010). Identification and characterization of genes responsible for biosynthesis of kojic acid, an industrially important compound from *Aspergillus oryzae*. Fungal Genet. Biol. 47, 953–961. 10.1016/j.fgb.2010.08.014 20849972

[B66] Torres A. M. Barros G. G. Palacios S. A. Chulze S. N. Battilani P. (2014). Review on pre-and post-harvest management of peanuts to minimize aflatoxin contamination. Food Res. Int. 62, 11–19. 10.1016/j.foodres.2014.02.023

[B67] Williams J. H. Grubb J. A. Davis J. W. Wang J. S. Jolly P. E. Ankrah N. A.. (2010). HIV and hepatocellular and esophageal carcinomas related to consumption of mycotoxin-prone foods in sub-Saharan Africa. Am. J. Clin. Nutr. 92, 154–160. 10.3945/ajcn.2009.28761 20484447

[B68] Williams J. H. Phillips T. D. Jolly P. E. Stiles J. K. Jolly C. M. Aggarwal D. (2004). Human aflatoxicosis in developing countries: a review of toxicology, exposure, potential consequences, and interventions. Am. J. Clin. Nutr. 80, 1106–1122. 15531656 10.1093/ajcn/80.5.1106

[B69] Williams W. P. (2006). Breeding for resistance to aflatoxin accumulation in maize. Mycotoxin Res. 22, 27–32. 10.1007/BF02954554 23605498

[B70] Wu F. (2015). Global impacts of aflatoxin in maize: trade and human health. World Mycotoxin J. 8, 137–142. 10.3920/WMJ2014.1737s

[B71] Yang L. Fountain J. C. Chu Y. Ni X. Lee R. D. Kemerait R. C. (2016). Differential accumulation of reactive oxygen and nitrogen species in maize lines with contrasting drought tolerance and aflatoxin resistance. Phytopathology 106, S2.16. 10.3390/ijms161024791 PMC4632777 26492235

[B72] Yang L. Fountain J. C. Wang H. Ni X. Ji P. Lee R. D.. (2015). Stress sensitivity is associated with differential accumulation of reactive oxygen and nitrogen species in maize genotypes with contrasting levels of drought tolerance. Int. J. Mol. Sci. 16, 24791–24819. 10.3390/ijms161024791 26492235 PMC4632777

[B73] Yu J. Chang P. K. Ehrlich K. C. Cary J. W. Bhatnagar D. Cleveland T. E.. (2004). Clustered pathway genes in aflatoxin biosynthesis. App. Environ. Microbiol. 70, 1253–1262. 10.1128/AEM.70.3.1253-1262.2004 15006741 PMC368384

