# Supplementary figures and images for: Responses of Aspergillus flavus to Oxidative Stress Are Related to Fungal Development Regulator, Antioxidant Enzyme, and Secondary Metabolite Biosynthetic Gene Expression
Source: Front Microbiol. 2016 Dec 21;7:2048. doi: 10.3389/fmicb.2016.02048 (PMC5175028; doi:10.3389/fmicb.2016.02048)

Supplemental Figure S1

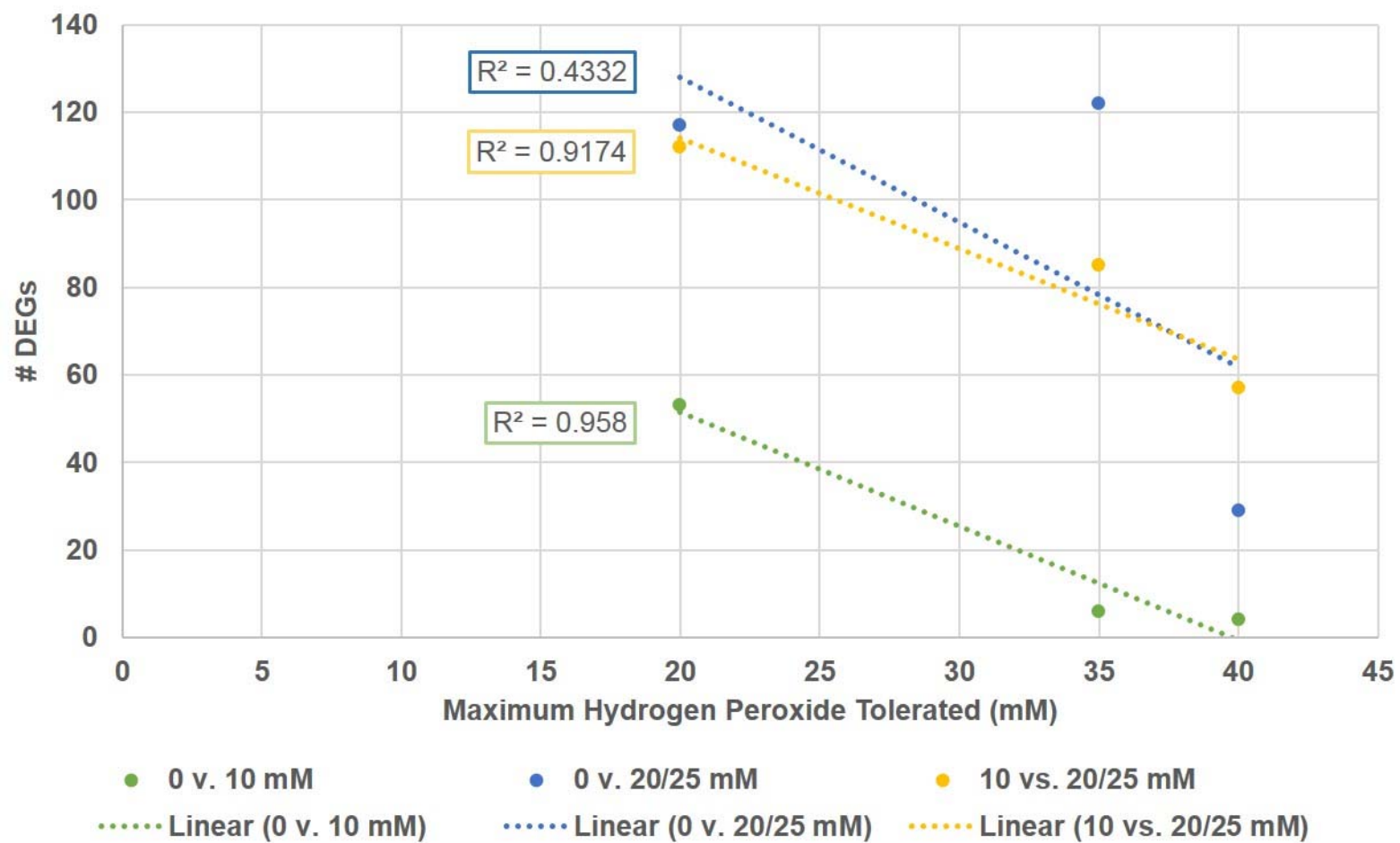

Supplemental Figure S2

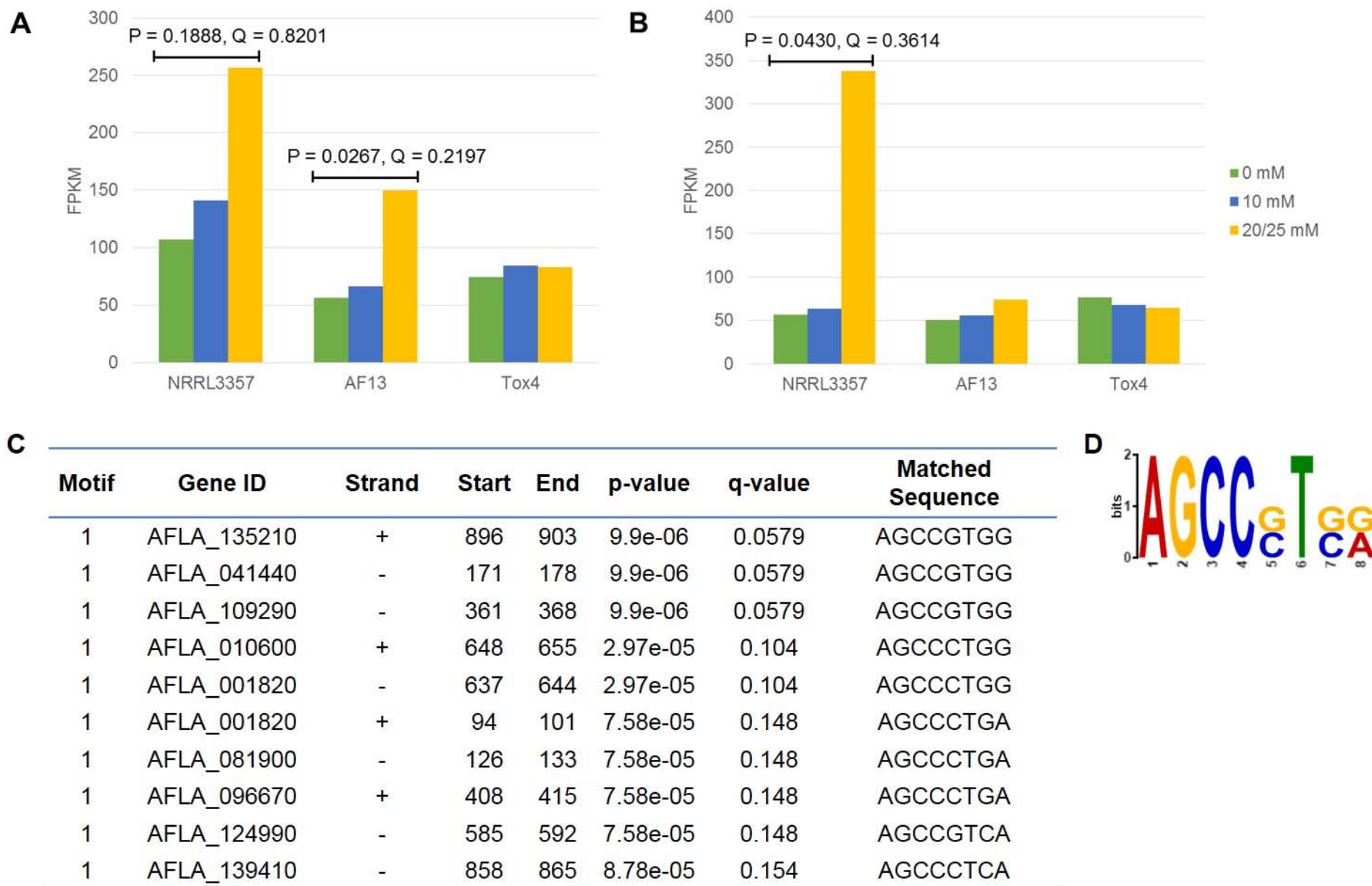

Supplement: Figure S1 — Linear regression analysis of the numbers of differentially expressed genes relative to isolate H2O2 tolerance. The numbers of significantly, differentially expressed genes were plotted relative to the observed maximum H2O2 level tolerated by the isolates observed in Fountain et al. (2015). Significant correlations were observed when comparing 0 and 10 mM H2O2 (R2 = 0.9580), and 10 and 20/2 5 mM H2O2 (R2 = 0.9174). The correlation between 0 and 20/25 mM H2O2 was not as significant (R2 = 0.4332) possibly due to a similarity between NRRL3357 and AF13 responses to high levels of stress. Numerical data can be found in Table 1. [file SupplementaryFigures.pdf]
